# Mitigation of Diabetes Mellitus Using *Euphorbia helioscopia* Leaf Ethanolic Extract by Modulating GCK, GLUT4, IGF, and G6P Expressions in Streptozotocin-Induced Diabetic Rats

**DOI:** 10.1155/2024/5497320

**Published:** 2024-09-18

**Authors:** Ahmed Raza, Muhammad Naveed Mushtaq, Sadia Hassan, Ali Sharif, Bushra Akhtar, Muhammad Furqan Akhtar

**Affiliations:** ^1^ Faculty of Pharmacy The University of Lahore, Lahore 54000, Pakistan; ^2^ Department of Biomedical Engineering and Sciences School of Mechanical and Manufacturing Engineering National University of Science and Technology, Islamabad 24090, Pakistan; ^3^ Department of Pharmacology Faculty of Pharmaceutical and Allied Health Sciences Lahore College for Women University, Lahore 54000, Pakistan; ^4^ Department of Pharmacy University of Agriculture, Faisalabad 38000, Pakistan; ^5^ Riphah Institute of Pharmaceutical Sciences Riphah International University Lahore Campus, Lahore 54000, Pakistan

**Keywords:** antioxidant, diabetes mellitus, *Euphorbia helioscopia* Linn, polymerase chain reaction, streptozotocin

## Abstract

Diabetes mellitus is a metabolic disorder. Synthetic antidiabetics are the commonly used treatment options associated with complications. The objective of this study was to explore the antioxidative and antidiabetic potential of *Euphorbia helioscopia* whole plant ethanolic extract using in vitro and in vivo models. For that purpose, the antioxidative potential was explored by using 2,2-diphenyl-1-picrylhydrazyl analysis. In vitro antidiabetic potential of the extract was evaluated using amylase inhibitory analysis. In vivo antidiabetic activity of the extract was assessed in diabetic rats using streptozotocin/nicotinamide (60 mg/kg/120 mg/kg) as an inducing agent. Metformin was used as standard. The results indicated the presence of significant quantities of phenolic 82.18 ± 1.28 mgg^−1^ gallic acid equivalent (GAE) and flavonoid 66.55±1.22 mgg^−1^ quercetin equivalent (QE) contents in the extract. Quantitation of phytoconstituents exhibited the presence of sinapic acid, myricetin, and quercetin using HPLC analysis. The extract inhibited *α*-amylase by 84.71%, and an antiglycemic potential of 50.34% was assessed in the OGTT assay. Biochemical analysis demonstrated a reduction in urea, creatinine, cholesterol, low-density lipoprotein, and alkaline phosphatase (*p* < 0.001) as compared to diabetic control rats at the dose of 500 mg/kg. An upregulation in the expressions of glucokinase, glucose transporter 4, peroxisome proliferator-activated receptor *γ*, and insulin-like growth factor was observed in treated rats in contrast to G6P expression, which was downregulated upon treatment. In conclusion, this study provided evidence of the antioxidative and antidiabetic potential of *E. helioscopia* whole plant ethanolic extract through in vitro and in vivo analysis and emphasized its promising role as a natural alternative.

## 1. Introduction

Diabetes is a chronic disease that silently causes life-altering morbidity and exacerbates many other complications due to its heterogeneity, and yet the reason lies in a single blood-borne metabolite, glucose. The rate of incidences of diabetes has been increasing since the 1980s, as statistics have shown that there were 108 million cases of diabetes in 1980 [1], which increased by the rate of 3% and reached 422 million people in 2014 [1] and 529 million in 2021 globally [2], and by 2045, it is expected to make 693 million people suffer [3]. Diabetes is a complex multisystemic metabolic disorder that is characterized by having higher concentrations of glucose in the blood due to the inability or insensitivity of pancreatic *β*-cells, resulting in inadequate insulin levels in the body.

There is a myriad of complications that arise due to higher glucose levels in the body, and it affects all the organs, including the heart, kidneys, nerves, and blood vessels. The malfunctions in these organs are associated with abnormalities in carbohydrate, fat, and protein metabolism, which are disturbed due to imbalanced glucose and insulin levels. The levels of insulin are important for human cells, especially skeletal muscle and adipocytes [4]. The most common type of diabetes is Type 2 diabetes mellitus (T2DM), which is noninsulin-dependent DM and currently affects 90% of the patients who have been diagnosed with diabetes [5, 6].

Due to the higher rate of complications, diabetes has become a major issue in the world. Antidiabetic drugs have become a major part of the pharmaceutical industry, as the global expense of antidiabetic drugs in 2021 was $966 billion. Forecasting data has shown that it is expected to go up to $1054 billion in 2045 [7]. The major players in fighting diabetes include sulfonylureas, meglitinides, biguanides, thiazolidinediones, and *α*-glucosidase inhibitors, and famous antidiabetic drugs from these groups are glipizide, glimepiride, repaglinide, metformin, rosiglitazone, acarbose, voglibose, and many others. Due to the longer-term use of synthetic drugs, a few complications have been reported in different studies; that is, a few studies reported the issues of clinical neuropathy and vitamin B12 deficiency associated with metformin and blood cancer with rosiglitazone [8]. In the current era, phytotherapy has become a major field. It is being widely used across the world to find new active sources from nature to cure heterogenic and chronic diseases [9]. The reason for using medicinal plants is their safety, quality, and effectiveness. They have been found to have lower toxicity profiles and high patient compliance, which makes them a suitable candidate and a good source of innovative pharmaceuticals [10].

Many plants in nature have strong antioxidant and antidiabetic properties. Among these plants, the Euphorbiaceae family has been associated with good antioxidative [11], antidiabetic [12], anticancer [13], and anti-inflammatory [14] properties. It has been observed that plants from the Euphorbiaceae family have the potential to reduce hyperglycemia and normalize blood glucose levels [15], as well as improve the lipid profile and control cholesterol levels in the body [16]. Among many plants from this family, *Euphorbia helioscopia* Linn (*E. helioscopia*) is a prominent herbaceous plant that has been used as a medicinal plant due to its extensive therapeutic properties. Due to the presence of strong antioxidants, it was used to fight oxidative stress-induced diseases, that is, diabetes. In another study, Sharma et al. [17] reported the anti-inflammatory and anticancer potential of *E. helioscopia*. In some other studies, antibacterial potential was also reported [18].

In many studies, antidiabetic and antioxidative potentials of the Euphorbiaceae family were reported; that is, Mustafa et al. prepared different extracts (methanol, ethanol, and aqueous) and reported preliminary data to demonstrate the antidiabetic and antioxidative potential of this family [19]. In a nutshell, the antidiabetic and antioxidant potential of *E. helioscopia* has been reported and discussed in the literature; however, a clear relationship between the antioxidative and antidiabetic potential at the macrolevel and molecular levels has never been studied. In this study, the antioxidative potential of *E. helioscopia* was explored, and antioxidative components were identified. Then, the antidiabetic potential was explored by using an extensive set of experiments with both healthy and diabetic animals. In addition, both acute and chronic glucose levels were assessed, and their effects on multiple biological moieties and animal tissues were observed. In the end, the molecular basis of the effects of *E. helioscopia* was evaluated on different functional and apoptotic genes. In conclusion, this paper offers a comprehensive study regarding the antioxidative and antidiabetic potential of *E. helioscopia* extract. It provides a clear link between treatment regimens and their effects on diabetic animals at both tissue and molecular levels using in vitro and in vivo models.

## 2. Methods and Materials

### 2.1. Chemicals

The fresh leaves of *E. helioscopia* were collected in April 2018 from the city of Kasur, and taxonomic identification and authentication were done (Voucher # GC.Herb.Bot.3465) by Professor of Botany, Dr. Zaheer-Uddin from Government College University, Lahore. The materials for analysis, including ascorbic acid, acarbose, nicotinamide, 2,2-diphenyl-1-picrylhydrazyl (DPPH) reagent, streptozotocin (STZ), and *α*-amylase, were obtained from Sigma-Aldrich (Germany). Other chemicals, including a TRIzol reagent, were provided by Advance Bioscience (Germany). All the materials were of analytical grade. Lastly, the kits for biological analysis were obtained from Bio-Labs (Pakistan) and Vivantis Technologies (Malaysia). Approval was obtained from the Institutional Research Ethics Committee (IREC-2018-45) of the University of Lahore for the performance of animal studies.

### 2.2. Preparation of Plant Extract

The first step was to prepare the ethanolic extract of the *E. helioscopia* plant, and for that purpose, instructions from Mousavi et al. [20] were followed. Briefly, 5 kg of the fresh plant was taken, thoroughly washed, and dried to eliminate the dust particles. Then, the plant was diced into small pieces and left for air-drying for 21 days in a protected environment. Subsequently, the plant material was crushed to obtain fine powder, and 420 g of the dry powder was obtained, which was macerated in 98% ethanol for 7 days. The resultant solution was filtered with Whatman No. 1 filter paper. The filtrate was subjected to high-pressure conditions in a rotary evaporator at 40°C to obtain a concentrated extract. Finally, the concentrated filtrate was overdried at 40°C, and the resulting powdered extract was stored at 4°C for further analysis. The percentage of yield for the extract was calculated using Equation (1).

(1) $$\begin{array}{c} \text{Percentage yield}=\frac{\text{Actual yield}}{\text{Total amount of powdered plant }}\times 100 \end{array}$$

### 2.3. Preliminary Phytochemical Screening

The extract of *E. helioscopia* was subjected to different analyses for phytochemical screening to explore its antioxidant content. For that purpose, two tests were performed, that is, total phenolic content (TPC) and total flavonoid content (TFC). The Folin–Ciocalteu reagent was used to determine the TPC *E. helioscopia* extract, and instructions from Khan et al. [21] were followed with minor modifications. Briefly, different concentrations of *E. helioscopia* extract were prepared with the help of ethanol. Then, in 9 mL of each concentration, 1 mL of Folin–Ciocalteu's reagent was added and mixed for 5 min, which was termed Solution A. At the same time, 10 mL of 7% sodium carbonate solution was mixed with 4 mL water. Finally, this mixture was added to Solution A and incubated at 37°C for 1.5 h. The concentration of each sample was measured using an ultraviolet (UV) spectrophotometer (Shimadzu UV-1700, Suzhou Instruments Manufacturing Company Limited, Suzhou, China) at 760 nm wavelength. The TFC was assessed through an aluminum chloride colorimetry assay by following the instructions from Zafar et al. [22]. To explain briefly, a 200 *μ*L extract dilution was prepared, and then an AlCl_3_ (100 *μ*L) and 1 M potassium acetate (100 *μ*L) were added, and the volume was raised to 5 mL with the help of distilled water. At the same time, a quercetin calibration curve was prepared using a UV spectrophotometer (K9000 UV, Shanghai Yoke Instrument Co. Ltd.) at 415 nm wavelength.

### 2.4. In Vitro Antioxidant Activity by DPPH

The objective of this test was to determine the scavenging capability of the extract using a DPPH scavenging assay. DPPH solution was prepared by dissolving 12 mg of DPPH powder in methanol. The solution was kept in a dark bottle to avoid the interactions of light. After 30 min, 1 mL of extract was combined with 2 mL of DPPH solution and analyzed through a UV spectrophotometer (Shimadzu UV-1700, Suzhou Instruments Manufacturing Company Limited, Suzhou, China) at 517 nm wavelength. The following equation was used to calculate the percentage inhibition capability of the extract.

(2) $$\begin{array}{c} \text{Inhibition}\%=\left[\frac{\text{Absorbance }\left(\text{control}\right)-\text{absorbance }\left(\text{sample}\right)}{\text{Absorbance }\left(\text{control}\right)}\right]\times 100 \end{array}$$

Ascorbic acid was used as a positive control solution, and methanol was used as a negative control. The analysis was done using different concentrations of the extract to calculate IC_50_ values [23]. The standard used was ascorbic acid.

### 2.5. Quantification of the Antioxidative Agents

After the confirmation of the presence of flavonoids and phenolics in the drug extract, high-performance liquid chromatography (HPLC) was performed to identify components. For this purpose, the LC-20A liquid chromatography system (Shimadzu, Japan) was utilized, which had an LC-10AT pump and PDA detector SPD-10AV. For detection purposes, a C18 column with dimensions of 25 cm × 4.6 mm × 5 *μ*m was used. The mobile phase and other conditions were optimized using the instructions from Malik, which were 280 nm detection wavelength, flow rate 1 mL/min, and a mixture of Solution A (H_2_O: acetic acid [94:6]) and Solution B (acetonitrile 100%) as mobile phase [24]. The chromatographs were obtained from the inbuilt software of the HPLC system.

### 2.6. Antidiabetic Potential Analysis

It was hypothesized that *E. helioscopia* extract has antidiabetic potential, so two tests were performed. The details are explained in upcoming sections.

#### 2.6.1. *α*-Amylase Inhibitory Activity

To determine the hypoglycemic ability of *E. helioscopia*, *α*-amylase inhibitory assay was performed. For that purpose, instructions from Khan et al. [21] were used. Amylase solution, the starch solution (0.5%), and phosphate buffer solutions (0.02 M) were prepared. Then, 1 mL of extract was taken in a test tube, and 1 and 2 mL of amylase solution and phosphate buffer were added, respectively, and incubated for 15 min at room temperature. Afterward, the starch solution was added and mixed thoroughly for 15 min. Then, this solution was put in a heating batch at 90°C temperature, and 3,5-dinitrosalicylic acid was added. It was incubated for 8 min. Subsequently, the solution was obtained, and UV absorbance was assessed at 540 nm using a UV spectrophotometer (Shimadzu UV-1700, Suzhou Instruments Manufacturing Company Limited, Suzhou, China). The percentage inhibition was calculated using the following equation. Acarbose was used as a control.

(3) $$\begin{array}{c} \text{Inhibition}\%=\left[\frac{\text{Absorbance }\left(\text{control}\right)-\text{absorbance }\left(\text{sample}\right)}{\text{Absorbance }\left(\text{control}\right)}\right]\times 100 \end{array}$$

#### 2.6.2. Oral Glucose Tolerance Test (OGTT)

The purpose of this test was to confirm the antidiabetic potential of *E. helioscopia* extract, and for that purpose, an albino rat model was selected [25]. To perform this test, 25 animals were recruited and randomly divided into five groups. After recruitment, the animals were kept under observation for 5 days to ensure their healthy status. The details of animals are given in detail in Table 1. On the day of the experiment, the groups were labeled and their weights were recorded. Then, animals were given food (carbohydrate 58%, fat 5.7%, protein 24%, ash 8%, and fiber 6%) and treatment dosages, which are explained in Table 1. Group I was labeled as a control group and was given 2 g/kg of glucose orally mixed with normal feed. The second group was labeled as a positive control group and was given metformin 100 mg/kg. The remaining groups were experimental groups, and each was given different amounts of *E. helioscopia* extract. The positive control and experimental groups were given treatments after 5 min of feeding the glucose. The blood samples were obtained from the tails of the animals at different time points (0, 30, 60, 90, and 120 min), and glucose levels were measured using an Accu-Check glucometer (Roche, Switzerland). The hypoglycemic potential of the plant was determined by calculating the area under the curve (AUC) on GraphPad Prism 5.0.

From this test, not only were glucose levels measured but also the percentage of inhibition of treatment agents was evaluated. The following equation was used for the percentage inhibition calculation.

(4) $$\begin{array}{c} \%\text{Hyperglycemia inhibition factor}=\frac{\text{AUC }\left(\text{control group}\right)-\text{AUC }\left(\text{treatment group}\right)\;}{\text{AUC }\left(\text{control group}\right)\;}\times 100 \end{array}$$

### 2.7. Animal Studies

After getting the confirmation of the antidiabetic potential of the proposed drug extract, further analysis was done on diabetic rats to observe the effects of the drug on diseased animals. For that purpose, different tests were performed to analyze the antidiabetic effects of *E. helioscopia* extract on diseased animals. The details of tests are explained in successive studies.

#### 2.7.1. Induction of Disease

Thirty healthy animals (*Rattus norvegicus*) 90-day-old weighing were recruited and observed for 5 days to find any issue, disease, or anomaly in animals. The rats were fed with normal rat chow (carbohydrates 43%, proteins 17%, and fats 40%) obtained from Hi-Tech feeds Lahore and tap water ad libitum. Animals were randomly divided into six groups, and each group was comprised of five animals. For the induction of diabetes, rats were subjected to fasting conditions for 12 h. Then, the animals were injected intraperitoneally with a single dose of nicotinamide (120 mg/kg). After waiting for 15 min, the rats were injected with a single dose of 5% dextrose solution and then with STZ (60 mg/kg) injection. The confirmation of diabetes induction was confirmed by checking the blood glucose levels. For the next 21 days, each group except for the first group (NC) was given a specific food and drug dosing, which are explained in Table 2. The dose solution was given with the help of an oral gavage tube.

On the 21st day of the study, 12 h fasted rats were anesthetized, blood was collected through the cardiac puncture, and the rats were humanely euthanized for collection of the pancreas. The antihyperglycemic potential of *E. helioscopia* extract was analyzed in rats and compared with the group with the commercially available antidiabetic drug metformin. There were six groups of animals, and each group contained five rats. The details of labeling and division are given in Table 2. The doses were selected on the basis of an existing study claiming *E. helioscopia* is safe up to the dose of 2000 mg/kg [26].

The conditions for animals were maintained for 21 days, and their glucose levels and weight variation were recorded at regular intervals [22]. In addition, different tests were performed by obtaining blood and tissue samples from animals.

#### 2.7.2. Antihyperglycemic Analysis

For the antihyperglycemic analysis, blood samples were obtained from each animal on the last day of the experiment, and glucose levels were monitored using the Accu-Chek Advantage II Clinical Glucose meter. To explain briefly, the blood was obtained from the tips of the tails of rats. The blood was placed on the tip of the glucometer, and the glucose concentration value was monitored [27].

#### 2.7.3. Weight Variations

The objective of this test was to analyze the effects of diabetes and our treatment regimens on the weights of animals. For that purpose, weight was monitored at the start and end of the study [28].

#### 2.7.4. Biochemical Analysis

For biochemical analysis, three types of testing were performed to observe the effects of *E. helioscopia* extract on different biological entities, including liver function tests, renal function tests, and lipid profiling tests. For that purpose, different chemical entities including triglyceride contents, cholesterol contents, high-density lipoprotein level (HDL), low-density lipoprotein level (LDL), very low-density lipoprotein level (v-LDL), alanine aminotransferase level (ALT), alkaline phosphatase level (ALP), aspartate aminotransferase level (AST), urea concentration, and creatinine contents were analyzed and their concentrations in the blood were measured.

Blood was obtained from animals on the 21st day to perform these tests. Briefly, the rats were given anesthesia, and then cervical dislocation was performed by restraining the heads of the animals and pushing them forward and downwards. The lower body of the animal was pulled backward by holding it from the tail base. The blood was collected from the heart and stored in small tubes. For further analysis, blood samples were centrifuged at 1500 rpm for 15 min [29]. For lipid profiling, LipidPro and CheKine Alanine Aminotransferase (ALT/GPT) Activity Colorimetric Assay Kit were utilized. For renal function analysis, ABL90 FLEX PLUS (Radiometer, United States) was used. The data was collected and analyzed for statistical significance, and graphs were plotted.

#### 2.7.5. Histopathological Analysis

After completion of the study, the animals were sacrificed, and the pancreas was collected for histopathological analysis. For analysis, instructions from Uyar and Abdulrahman were followed [30]. Concisely, the pancreas was collected and washed with cold 0.9% saline. Afterward, formalin (10% neutral buffered solution) was used for tissue fixation. For further processing and dehydration of tissues, ethanol was used, and samples were fixed in paraffin wax. Finally, the tissues were cut into 5 *μ*m thick sections and stained with hematoxylin and eosin (H&E). For microscopic analysis, a light microscope (Optika, Ponteranica, Italy) was used, and it was equipped with an optical camera that makes linear measurements. The results were analyzed and prepared by a certified pathologist to minimize human error [29].

### 2.8. Analysis of Molecular Gene Expressions

Once the suitable candidate was selected through animal studies, the effects of *E. helioscopia* extract on genetic expression were evaluated. For that purpose, reverse transcriptase RT-PCR was used. To perform the analysis, ribonucleic acid (RNA) samples were isolated from pancreatic tissues with the help of a TRIzol reagent. Then, the protocol described by the complementary deoxyribonucleic acid (cDNA) synthesis kit (Cat No. 4368814) was used to reverse transcribe the RNA sample. The primers were designed and synthesized for further analysis.

After obtaining cDNA, it was amplified in a thermal cycler, and for that purpose, PCR (Cat No. 32161000) was used. All the instructions provided by the manufacturer were followed to obtain the amplified samples. In the meantime, the preparation of agarose gel running was completed. The samples were stained with ethidium bromide and run on gel. Once the electrophoresis was done, the pictures and data were collected and analyzed through different software to get quantifiable results [31–33].

### 2.9. Data Analysis

The data was presented by taking the mean ± SD. GraphPad Prism 5.0 was used to determine IC_50_ using a nonlinear regression model. Furthermore, one-way analysis of variance (ANOVA) was also used to analyze the data, followed by Bonferroni's multiple comparison test. The electrophoretic picture was quantified by ImageJ software and GraphPad Prism 5.0.

## 3. Results and Discussion

After obtaining the extract from *E. helioscopia*, a percentage yield was calculated, which was found to be 2.94%. These results were in line with the results of Maoulainine et al. [34]. In the literature, different solvents have been recommended for *E. helioscopia*, that is, methanol, ethanol, and chloroform; however, in this study, ethanol was used due to its better extractive abilities, sample-to-solvent ratio, and extraction conditions. After confirming the yield, a different analysis was performed, and their details are given below.

### 3.1. Preliminary Phytochemical Screening

The objective of this test was to confirm the presence of phenolic and flavonoid compounds in the extract. The results of TPC and TFC are demonstrated in Table 3, which shows that the *E. helioscopia* extract has 82.18 + 1.28 mgg^−1^ gallic acid equivalent (GAE) phenolic content and 66.55 + 1.22 mgg^−1^ quercetin equivalent (QE) flavonoid content.

It has been found that higher flavonoid and phenolic content increases the antioxidant, anti-inflammatory, and anticancer potential of drugs and medicines [35]. Usually, plant extracts show higher flavonoid and phenolic content; that is, in a study, *Zanthoxylum armatum* fruit was reported to have 22.8 ± 1.33 mg/g phenolic content. In the current study, *E. helioscopia* extracts demonstrated 82.18 mgg^−1^, which is in line with the results reported by Mustafa et al. [19]. In the literature, different studies have compared the phenolic content of different types of extracts of *E. helioscopia*, and the methanolic extract was found to have the highest amount of phenols and flavonoids [34].

### 3.2. Antioxidation Analysis

**Figure 1:**
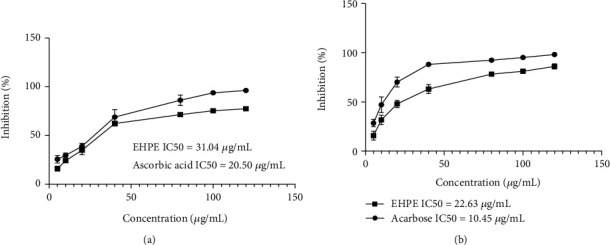
(a, b) Percentage inhibition of *E. helioscopia* whole plant extract: (a) DPPH assay and (b) alpha-amylase assay.

The presence of phenolics and flavonoids was confirmed, and this test was performed to measure the overall antioxidative potential of *E. helioscopia* extract. The results are exhibited in Figure 1(a), which shows a higher percentage inhibition at 120 *μ*g/mL (77.27%) with IC_50_-31.04 *μ*g/mL as compared to ascorbic acid, which was used as standard. Ascorbic acid demonstrated IC_50_-20.50 *μ*g/mL with a percentage inhibition (96.08%).

It has been observed that reactive oxygen species in the body promote the oxidization of different biological entities, that is, proteins, nucleic acids, and lipids; however, the presence of antioxidants helps to reduce oxidative stress in the body, which ultimately reduces the complications of diabetes [36]. As *E. helioscopia* has demonstrated a good potential for antioxidation, it may be beneficial for diabetic patients to control the symptoms of their disease.

### 3.3. Quantification of Antioxidants

The purpose of the HPLC analysis was to confirm the presence of antioxidants in plant extract and quantify their concentration. The results are presented in Table 4. There are three main components of *E*. *helioscopia* extract that may be responsible for the higher content of phenols and flavonoids.

According to Yang et al., *E. helioscopia* contains more than 170 components, including lipids, terpenoids, flavonoids, and phenols [37]. Quercetin is an important antioxidant that is responsible for its antioxidant properties. It is commonly found in grains, onions, grapes, and vegetable leaves. In the literature, quercetin has demonstrated protective effects in diabetic patients due to its ability to decrease oxidative stress and preserve the integrity of pancreatic *β*-cells [38]. Similarly, sinapic acid has been reported to promote mitochondrial biogenesis by upregulating the oxygen consumption rate. In addition, it enhanced the expression of peroxisome proliferator-activated receptor *γ* coactivator-1*α* (PGC-1*α*) and UCP1 [39]. Myricetin is an important component of plants that imparts antioxidant properties. It belongs to polyphenolic compounds and has been reported to have antihyperglycemic properties [40]. This compound helps in the regulation of glucose transport through the GLUT2 pathway and is linked with increased insulin sensitivity and inhibition of pancreatic *β*-cell apoptosis [41]. In conclusion, the presence of sinapic acid, myricetin, and quercetin confirms the increase in glucose uptake and glycogen synthesis in the body, which will, in return, improve the glucose balance and lipid profiles. It will ultimately lead to a decrease in complications and adverse reactions in the body [42].

### 3.4. Antidiabetic Potential Analysis

This study hypothesized that *E. helioscopia* extract has antidiabetic potential, so two tests were performed. The details are explained in upcoming sections.

#### 3.4.1. *α*-Amylase Inhibitory Assay

The amylase inhibition capability of *E. helioscopia* extract was evaluated using *α*-amylase inhibitory assay, and results are demonstrated in Figure 1. It was found that *E. helioscopia* extract had 85.9% (IC_50_-22.63 *μ*g/mL) percentage inhibition of *E. helioscopia* (Figure 1(b)). Surprisingly, the percentage inhibition was close to the percentage inhibition of acarbose at 98% (IC_50_-10.45 *μ*g/mL). A change in the absorption process of glucose was confirmed by a reduction in the AUC.

Studies have shown that the presence of phenolics and flavonoids increases the amylase inhibition action by binding themselves with proteins to inhibit glucoside hydrolases [24]. In this study, the *E. helioscopia* extract demonstrated significant inhibition of hyperglycemia in rats, and supervising, it was equivalent to the percentage inhibition of metformin. The results corroborated the previous findings reported in the literature [43].

#### 3.4.2. OGTT

**Figure 2:**
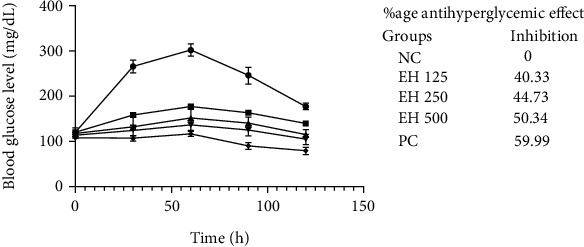
Blood glucose levels for different groups of animals through the glucose tolerance test.

The OGTT is performed to analyze the body's response to glucose tolerance, and it is used as a screening test for diabetes detection. The results of this study are exhibited in Figure 2, which shows that *E. helioscopia* has excellent antiglycemic potential, which is 50.34% as compared to the control drug (59.99%). In addition, this test gave us excellent information about the effects of different treatment regimens on blood glucose levels over time. All the treatment arms showed that as soon as rats ingested the food, the glucose levels started to elevate; however, the concentration of glucose levels for treatment rats did not have a sharp rising peak. Instead, it rose gradually and at a lower rate than the rising levels of normal control rats. The interesting thing about positive control was that it did not rise at all and started to decline after 1 h. Nevertheless, after 2 h, for higher concentrations of *E. helioscopia,* the glucose level was even lesser than levels at 0 min. The rate of the decline of glucose levels was more prominent in EH250 rats.

It was observed that the higher the concentration of *E. helioscopia*, the higher would be the blood glucose inhibition capability. The Euphorbiaceae family is associated with multiple types of metabolites, which are responsible for the antiglycemic, antidiabetic, anti-inflammatory, and antioxidant-related properties. Similarly, in another study, the antidiabetic activity of *Uapaca bojeri* Bail (Euphorbiaceae) was exhibited [44]. The literature complements the results of our study, as Mustafa et al. and Widharna et al. have reported the antidiabetic and hypoglycemic potential of *E. helioscopia* extract [45, 46].

#### 3.4.3. Animal Studies

Once the antioxidative and antidiabetic ability of *E. helioscopia* extract was confirmed through preliminary studies, further analyses were conducted in diabetic animals to observe the antidiabetic activity and confirm its potential to be used as a treatment option for diabetes mellitus.

#### 3.4.4. Antihyperglycemic Analysis

**Figure 3:**
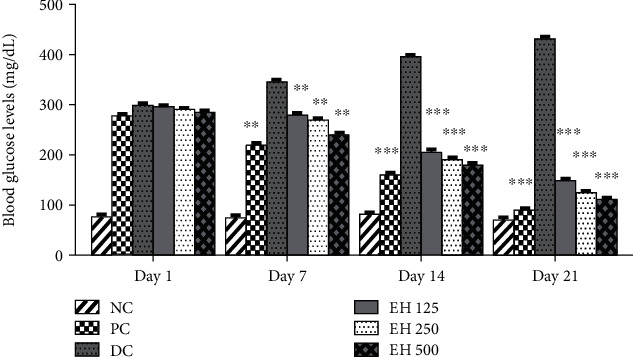
Blood glucose levels of different groups of animals in specific periods show the effects of various treatment options for the disease. ^∗^*p* < 0.05, ^∗∗^*p* < 0.01, and ^∗∗∗^*p* < 0.001 when compared to the diabetic control.

The antidiabetic effects of *E. helioscopia* extract were analyzed in rats, and the results are demonstrated in Figure 3. The experimental groups exhibited significant hypoglycemic potential in streptozotocin-nicotinamide (STZ-NA)-loaded rats. On the 1st day, the glucose levels of EH500 were 285.4 ± 2.07 as compared to the positive control group PC 278.2 ± 3.42 mg/dL. These levels declined significantly (*p* < 0.05) to 111.8 ± 3.70 and 90.6 ± 4.03 mg/dL on Day 21 (Figure 3). However, the glucose levels of diabetic control on the 1st and 21st days were 299.4 ± 3.04 and 460.4 mg/dL, respectively.

These results indicated the effectiveness of reducing the blood glucose levels in diabetic rats. It also showed that increasing the extract concentration reduced the blood glucose more effectively.

#### 3.4.5. Effect on Weight Alterations

The objective of this analysis was to observe the effects of diabetes and its treatments on the weight of the animals. The results are exhibited in Table 5, which shows that *E. helioscopia* extract had a positive impact on their health and helped to increase their appetite, leading to weight gain. In comparison to the normal group, the EH500 and other groups showed significantly higher (*p* < 0.05) weights.

Variance in weight is a commonly observed phenomenon in diabetic animal models, and weight is generally decreased in diabetic rats [47]. Nevertheless, the rats treated with *E. helioscopia* showed an increase in weight, which may be due to the animals' increased metabolic activity.

#### 3.4.6. Biochemical Analysis

Diabetes affects not only the blood glucose levels but also other biological entities, that is, urea, creatinine, cholesterol, and triglycerides; therefore, several biochemical analyses were performed. The details of each test are given below.

##### 3.4.6.1. Liver Function Tests

**Figure 4:**
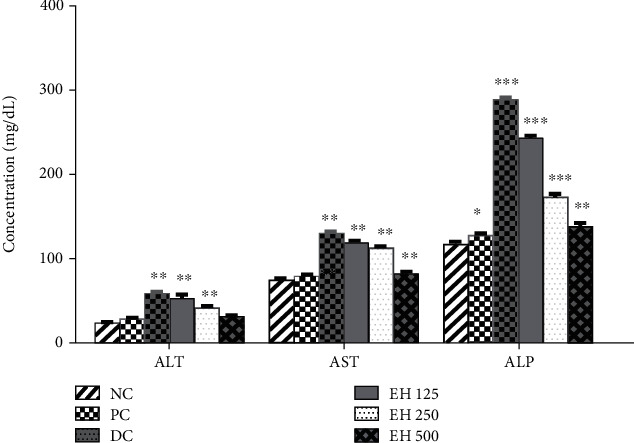
The effects of different treatment options on liver functions of diseased animals. ^∗^*p* < 0.05, ^∗∗^*p* < 0.01, and ^∗∗∗^*p* < 0.001 when compared to the normal control group.

The effects of *E. helioscopia* extract on treated animals were analyzed by performing different types of tests, including ALT, AST, and ALP, and the effects on liver functions are shown in Figure 4. A significant improvement in liver function was observed by the consumption of *E. helioscopia* extract. The value of diabetes control for the ALP test was 289 ± 2.73 mg/dL, which was significantly decreased by *E. helioscopia* extract, particularly by EH500, for which ALP values decreased to 138 ± 3.67 mg/dL. The other sample also significantly improved liver functions. In the literature, Saleem et al. have studied the effectiveness of *E. helioscopia* extract on liver functions in two different studies and reported improvement in ALP functions plant [26, 48].

Overall, EH500 was found to be more effective as compared to EH250 and EF125. A similar trend was observed in ALT and AST analysis, and all the samples were found to be effective; all three tests demonstrated effectiveness in a dose-dependent fashion, which may be attributed to the amelioration of hepatic phosphatases and transaminases related to decreased oxidation [24]. A decrease in ALT was observed by using *E. helioscopia* extract, which may be related to the improvement of insulin activity that resulted in a reduction of transcription of ALT-associated gluconeogenesis [49]. Lastly, the elevated urea level due to diabetes was also restored to normal after treatment with *E. helioscopia.*

##### 3.4.6.2. Renal Function Tests

**Figure 5:**
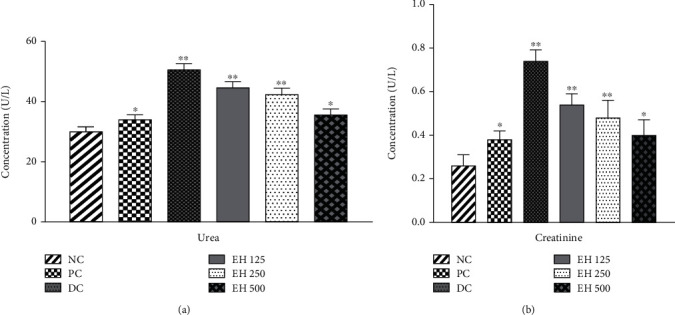
The effects of different treatment options on the renal function of the animals: (a) effects on the concentration of urea and (b) effects on the concentrations of creatinine. ^∗^*p* < 0.05, ^∗∗^*p* < 0.01, and ^∗∗∗^*p* < 0.001 when compared to the normal control group.

Renal function testing helps to understand the effectiveness of the body in removing toxins from the kidneys. In this study, analysis was performed to assess the removal of creatinine and urea from the body. The results are exhibited in Figure 5, which shows that *E. helioscopia* can reduce the concentration of both urea and creatinine in the body. The concentration of urea and creatinine was found to be 30.0 ± 1.58 and 0.26 ± 0.05 U/L, respectively, in normal rats, which increased to 50.60 ± 2.07 and 0.74 ± 0.05 for diabetic control; nevertheless, all the samples of the extract were found to be effective and reduced the levels of both chemicals in the body. It was also observed that increasing the concentration of the drug has an inverse relationship with both chemicals; that is, the amount of urea for all three extracts (EH125, EH250, and EH500) was 44.60 ± 2.07, 42.40 ± 2.07, and 35.60 ± 1.94, respectively. A similar trend was observed in creatinine analysis, as the amount was found to be 0.54 ± 0.05, 0.48 ± 0.08, and 0.40 ± 0.07 U/L, respectively.

The levels of urea and creatinine are important biomarkers for the identification of renal failure as they affect the glomerular filtration rate, and their elevated levels indicate nephrotoxicity. If the metabolism of animals is regulated, the salt levels will be balanced [50]. All the groups treated with *E. helioscopia* demonstrated lower levels of urea and creatinine, which shows that this plant has a good impact on the metabolism of animals and can regulate their metabolism. The *Euphorbia* genus has been reported in the literature as an important medicinal plant to improve renal functions. Liu, Zeng, and Hou studied the effectiveness of *Euphorbia pekinensis* in nephrotoxic rats and profiled its mechanism of action [51]. This was an important study because it provided evidence for toxic components and also provided a new reference to study nephrotoxins in traditional Chinese medicine.

##### 3.4.6.3. Lipid Profile Testing

**Figure 6:**
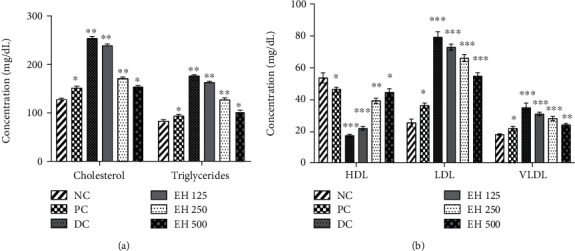
The effects of different treatment options on the lipid profile of the animals: (a) effects on the levels of cholesterol and triglycerides and (b) effects on the concentrations of LDL, HDL, and VLDL. ^∗^*p* < 0.05, ^∗∗^*p* < 0.01, and ^∗∗∗^*p* < 0.001 when compared to the normal control group.

Lipid profile testing helps to measure the lipid and fatty content in the body. The results of the serum lipid profile are exhibited in Figure 6, which explains that the treatment group had better LDL, HDL, and VLDL levels than the diabetic group. In addition, the rate of reduction of LDL and VLDL was dose-dependent, and the most effective composition was EH500, as it demonstrated 54.6 ± 2.19 and 23.8 ± 0.83 mg/dL, respectively, as compared to diabetic control, which shows 79.2 ± 3.34 and 34.6 ± 2.88, respectively. In addition, the concentration of cholesterol and triglycerides was significantly decreased by using *E. helioscopia*-based treatments.

It is known that diabetic patients usually possess lower levels of HDLs and higher levels of LDLs, and we observed this phenomenon in our study [52]. Plant sterols and other phytochemicals have a positive impact on the body and help to balance the cholesterol levels in the body; that is, in a study, Almalki, Alghamdi, and Al-Attar [53] reported different plants (*Olea oleaster* leaves, *Juniperus procera*, and *Opuntia ficus-indica)* for having the ability to reduce the cholesterol levels in rats. The results of our study indicated that *E. helioscopia* can maintain the cholesterol levels of the body and reduce the LDLs to avoid the complications of diabetes. This conjecture was complemented by the analysis of cholesterol levels, which were significantly reduced in diabetic rats by using *E. helioscopia* extract.

Effect of treatments on complete lipid profile. Data is presented as mean ± SD. The level of significance was estimated by one-way ANOVA followed by Fisher's LSD test and is expressed as ^∗^*p* < 0.05, ^∗∗^*p* < 0.01, and ^∗∗∗^*p* < 0.001 when compared to the normal control group. Abbreviations: NC, normal control; PC, positive control; DC+saline, diabetic rats treated with normal saline; EH, *E. helioscopia*; DC, diabetic control.

#### 3.4.7. Histopathological Investigations

**Figure 7:**
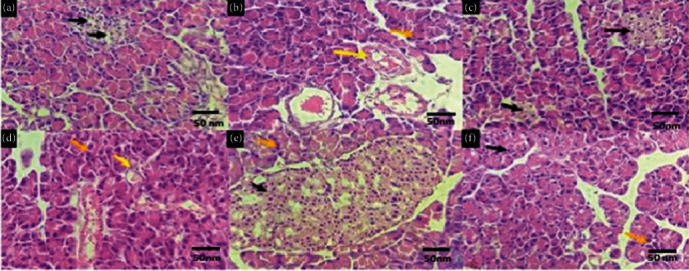
Histopathological analysis of different animals after receiving treatment. (a) Pancreatic tissues of normal animals (Group I). (b) Damage caused by diabetes in diabetic control animals (Group II). (c) Effects of metformin on diabetic animals (Group III). (d) Effects of the EH125 experimental sample on diabetic animals (Group IV). (e) Effects of the EH250 experimental sample on diabetic animals (Group V). (f) Effects of the EH500 experimental sample on diabetic animals (Group VI). Black arrows show inflammation, yellow arrows indicate the foci of beta-cell degeneration, and orange arrows exhibit acinar cells.

Figure 7(a) demonstrates a histological examination of the pancreatic tissue of a normal animal. The feature of this image revealed normal-looking endo- and exocrine elements of the pancreas. The islets of Langerhans contained normal-looking beta cell concentration, and the acinar cells appeared to have a normal appearance. In addition, no evidence of any degeneration, inflammation, calcification, granuloma, or malignancy was observed. In comparison, pancreatic tissue of diabetic control (Figure 7(b)) revealed normal-looking exocrine elements of the pancreas; however, there was an absence of islets of Langerhans. In addition, the foci of beta-cell degeneration could also be observed. A similar disappearance or shrinkage of islets of Langerhans due to diabetes has been observed by Saad et al. and Elkotby et al. [54, 55], which resulted in a decreased number of *β*-cells.

Nevertheless, metformin seemed to have a positive impact as it exhibited the presence of islets of Langerhans, which contained near the normal beta cell concentration (Figure 7(c)). The histological tests for metformin were similar to the results of a study by Balamash et al. in which metformin showed the absence of any degeneration and had a more or less normal number of islets of Langerhans [56]. Figure 7(d) represents the effects of EH125 on the diabetic rats and demonstrated recovered islets of Langerhans and normal-looking exocrine elements. Surprisingly, EH250 and EH500 had a good impact on recovery and showed almost normal pancreatic tissues. This showed that at higher concentrations, *E. helioscopia* extract has a better capability to achieve the desired therapeutic effects. These results were comparable to the previously reported studies in which different other plant extracts were used for antidiabetic activity and found to have a positive impact on pancreatic tissues [57].

Overall, a difference in the morphology of diabetic rats and normal rats can be observed through these images, and it proves that *E. helioscopia* extract may have the ability to treat the diabetic rats and restore them to their original form or bring them closer to the original morphology.

The histopathological examinations showed a dose-dependent improvement in the pancreas of STZ-induced diabetic rats when treated with *E. helioscopia* extract as compared to the disease control group. The findings of the current study corroborated the earlier investigation on herbal drugs, suggesting their role in the amelioration of oxidative damage in organs [22, 58].

#### 3.4.8. Molecular Studies

After the confirmation of the positive effects of *E. helioscopia* extract on diseased animals, EH500 was selected as the most effective concertation as it showed the best results among all the experimental specimens. Then, the effects of the EH500 were observed at the molecular levels to observe its effects on the upregulation and downregulation of different genes that are involved in diabetes and insulin resistance.

##### 3.4.8.1. Relative Gene Expression of Proliferative and Apoptotic Regulators

**Figure 8:**
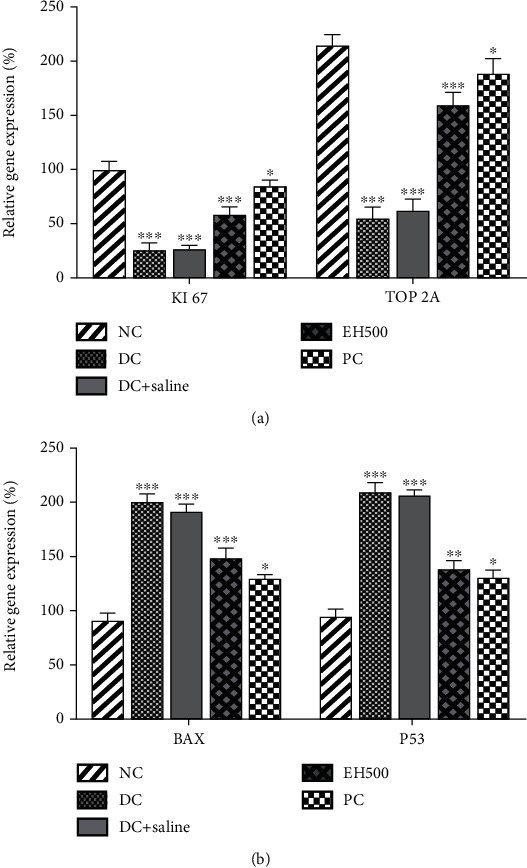
Effect of EH500 and metformin on relative mRNA expression of the genes: (a) effects on the genes related to apoptosis and (b) effects on the genes related to cell proliferation. ^∗^*p* < 0.05, ^∗∗^*p* < 0.01, and ^∗∗∗^*p* < 0.001.

The analysis of relative gene expression was done using samples from the pancreas of the animals, and the results are demonstrated in Figure 8(a). The gene expression was analyzed for KI67 and Topoisomerase 2 alpha (TOP2A). For both genes, a similar trend in gene expression was observed, which was NC > PC > EH500 > saline − treated > DC. The positive control showed gene expression closer to the normal range. Although EH500 could not show results equivalent to the normal range, it was closer to the normal range and was better than diabetic control.

The results were further confirmed by checking the expression of apoptotic regulators, that is, BAX and P53 genes, which are exhibited in Figure 8(b). The expression of the gene was found to be higher in diabetic rats; however, downregulation in expression was found in the treatment arms. The EH500 sample was able to downregulate both genes significantly, but the inhibition activity was less than positive control, leading to a comparatively higher rate of gene expression. Nevertheless, the downregulation of BAX and P53 genes confirms the antidiabetic and antioxidative potential of *E. helioscopia* extract.

The islets of Langerhans house pancreatic *β*-cells, which play an important role in controlling glucose levels by producing insulin. The reduction in pancreatic *β*-cells results in diabetes [59]. This reduction in *β*-cells is caused by apoptosis in the pancreas. In a prediabetic patient, pathological changes start to occur in *β*-cells, resulting in cell death due to exhaustion. The expression of KI67 is an important predictor of the evaluation of the proliferation of *β*-cells in pancreatic tissues [60].

Similarly, the Topoisomerase II enzyme (TOP2E) has been found to be associated with pancreatic cell proliferation [61]. In this study, the treatment arms demonstrated expression of KI67 and TOP2A closer to the expression that existed in normal animals. These results point out the normalcy of the pancreatic *β*-cells, as the diseased rats would have higher cell death rates and lesser proliferation rates. In addition, mRNA expression of KI67 is associated with the antioxidant activities of the drugs; thus, *E. helioscopia* extract was able to preserve the integrity of pancreatic *β*-cells and maintain tissue homeostasis and growth [62].

To explore further, the relative gene expression of apoptotic genes, that is, BAX and P53, was evaluated. BAX gene (Bcl-2-associated X-protein) is a proapoptotic regulator that codes for BAX-alpha protein. This protein is associated with Bcl-2 and causes induction of intrinsic apoptosis [63]. The results indicated that the use of *E. helioscopia* extract has less expression of BAX genes; therefore, it may be suggested that there was no cell degradation or apoptosis, and consequently, expression of apoptotic genes was not required. The situation was different in diabetic rats, as higher expression of BAX was causing the death of *β*-cells, which was reducing insulin production and causing the accumulation of the higher glucose levels in the body. P53 is responsible for the suppression of tumor growth; however, it plays a major role in metabolic activities, that is, glycolysis, *β*-oxidation, gluconeogenesis, and glycogen synthesis. In addition, its expression is important for insulin production and a reduction in glucose levels [64]. In this study, the expression of P53 was downregulated after treatment, which advocated a decline in oxidative stress and metabolic imbalance. The reason for the reduction of oxidative stress is correlated with the antioxidant properties of the drug, which have already been proven in previous experiments.

##### 3.4.8.2. Evaluation of Functional Genes

**Figure 9:**
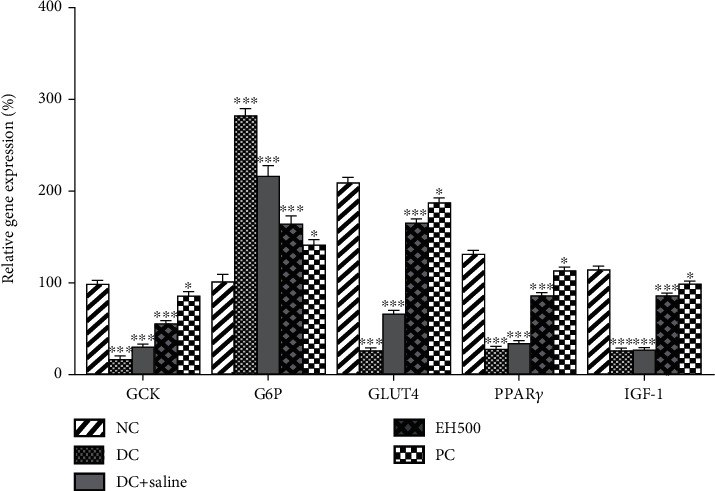
Effect of the treatments on relative mRNA expression of functional genes. ^∗^*p* < 0.05), ^∗∗^*p* < 0.01, and ^∗∗∗^*p* < 0.001.

There are a number of genes that are associated with glucose concentration and insulin levels in the body and are greatly affected by the onset of diabetes. This test was performed to analyze the effects of the EH500 sample on the expression levels of these genes and compare them with the expression levels of positive control and diabetic control. The results (Figure 9) show that among the five genes, all were upregulated in the treatment arm, and their expressions were similar to those of the normal control group except for G6P, which was downregulated. The downregulation of G6P is related to insulin-mediated actions, and if it is knocked down or downregulated, it indicates higher concentrations of insulin in the blood. On the contrary, the upregulation of the other four genes, GCK, GLUT4, IGF-1, and PPAR-*γ*, is associated with lower glucose levels; that is, GCK catalyzes the conversion of glucose into glucose-6-phosphate, and lower levels of glucose will indicate the higher levels of insulin in the body, which will cause the upregulation of this gene. Similar reasons are true for the other three genes. The experimental sample EH500 demonstrated its ability to control diabetes. Still, it had lesser antidiabetic effects as compared to the commercially available product Metformin, which has been termed a miracle drug for diabetic patients.

GLUT4 has an important relationship with insulin, as the higher the amount of insulin, the higher will be the concentration of GLUT4 [65]. The results showed the lowest concentration of glucose in diabetic rats because they have the highest concentration of glucose and the lowest concentration of insulin. The treatment arms (both positive control and EH500) demonstrated higher expression of GLUT4, which indicated the presence of higher concentrations of insulin in the metabolic systems of the animals. In addition, the results of our study were in line with Chen et al. [66], which reported the antiglycemic potential of *Chimonanthus nitens* leaf extract and demonstrated the upregulation of GLUT4 and GLUT1 in the treatment arms. Contrary to GLUT4, G6P has higher expression levels in diabetic rats. The reason behind this elevation in expression is the increased synthesis of the enzymes involved in glucose production during diabetes by the liver. Therefore, elevation in glucose levels is associated with higher rates of G6P expression [67]. In the treatment arm, *E. helioscopia* was able to downregulate the expression of this gene because of decreasing glucose levels in the body. A similar trend for G6P was observed in our previous study, in which a plant extract of the fig tree (*Ficus johannis* Boiss) was evaluated for its antidiabetic potential [68].

The gene called GCK plays a crucial role in diabetes diagnosis as this gene codes for protein “glucokinases,” which are known as “glucose sensors” in the body. It works oppositely to G6PC; however, their coordination regulates the free glucose in the body [65]. The lower levels of insulin result in a metabolism imbalance, which decreases the expression of GCK; nonetheless, the presence of antioxidants and antidiabetic agents helps to restore the metabolic balance in the body, which results in the upregulation of this gene. This also showed that treatment arms started to control the glucose concentration by using glycolysis, glycogenesis, and gluconeogenesis [69]. A similar trend was observed in the expression of IGF-1 and PPAR-*γ* genes. IGF-1, also known as somatomedin C, is one of the major regulators of lipid and glucose levels in the body. In the presence of higher glucose levels, its expression is downregulated, as we observed in our diabetic rat. However, balanced glucose and insulin levels improve lipogenesis and insulin sensitivity, which results in higher expression of IGF-1 [70].

Overall, the EH500 demonstrated promising results in regulating glucose concentration and insulin levels. It was not only able to downregulate BAX and P53 genes, which have apoptotic potential, but also upregulate Ki67 and TOP2A, showing the maintenance of the integrity of pancreatic *β*-cells. In addition, all the functional genes that regulate the blood glucose levels were effectively expressed and demonstrated good control over blood glucose and insulin levels. By looking at these results, it may be concluded that EH500 has the potential to decrease the glucose levels in the body and may be used as an alternative for commercial products.

## 4. Conclusion

The medicinal effects of *E. helioscopia* extract were reported in the literature, and this study provides an in-depth glimpse of the antioxidative and antidiabetic potential of this plant through different in vitro and in vivo studies. This can be attributed to the improvement in pancreatic functions, which is evident from the morphological evaluation of the pancreas. Quercetin, myricetin, and sinapic acid were quantified and may be responsible for the in vitro and in vivo antidiabetic potential. These flavonoids and phenolic acid may have exerted a potential role in the regulation of glucose by modulating GLUT4, G6P, IGF-1, and GCK in T2DM rats.

### 4.1. Limitations of the Study

The study can be improved by measuring serum insulin levels and applying a homeostatic model assessment for insulin resistance (HMOA-IR).

## Figures and Tables

**Table 1:** Details of the animals during experimentation.

**Groups**	**Group labels**	**Feed**	**Treatment**
I	NC	2 g/kg of glucose+normal feed	No treatment
II	PC	Metformin 100 mg/kg+normal feed	Commercially available drug
III	EH125	125 mg/kg extract+normal feed	Experiment drug
IV	EH250	250 mg/kg extract+normal feed	Experiment drug
V	EH500	500 mg/kg extract+normal feed	Experiment drug

**Table 2:** Details of the different types of treatment regimens.

**Sr no.**	**Group names**	**Group labels**	**Feed**	**Treatment**
1.	Normal control	NC	Normal food	Normal saline
2.	Positive control	PC		Metformin 100 mg/kg
3.	Diabetic control	DC		Normal saline by oral gavage
4.	Experiment Group II	EH125		125 mg/kg plant extract
5.	Experiment Group II	EH250		250 mg/kg plant extract
6.	Experiment Group III	EH500		500 mg/kg plant extract

**Table 3:** Total phenolic and flavonoid content in the extract.

**Sr #**	**Content name**	**Extract quantity**	**Quantitative analysis**
1	Total phenolic content	100 *μ*g mL^−1^	82.18 ± 1.28 mgg^−1^ GAE
2	Total flavonoid content		66.55 + 1.22 mgg^−1^ QE

**Table 4:** Quantification of phytoconstituents in the *E. helioscopia* ethanolic plant extract by HPLC.

**Compound**	**Retention time (min)**	**Width (min)**	**Area (mAU**∗**s)**	**Amount (ppm)**
Myricetin	4.158	0.2361	253.49	52.531
Quercetin	5.808	0.2530	19.163	4.781
Sinapic acid	4.485	0.1383	277.387	8.4582

**Table 5:** Effect of *E. helioscopia* ethanolic plant extract and metformin on body weight gain.

**Days**	**NC**	**PC**	**DC**	**EH125**	**EH250**	**EH500**
1	167.6 ± 1.14	169.4 ± 1.34	175.2 ± 1.92^∗∗^	173.6 ± 1.14^∗∗^	174 ± 1.58^∗∗^	176.4 ± 1.51^∗∗^
7	172 ± 1.58	174.2 ± 1.92	154 ± 2.54^∗∗^	177.2 ± 2.58^∗^	184.6 ± 3.13^∗∗^	186.2 ± 1.48^∗∗^
14	183 ± 3.16	178.2 ± 1.92^∗^	144.6 ± 2.30^∗∗^	187.8 ± 3.11^∗^	190.2 ± 3.27^∗∗^	192.2 ± 2.16^∗∗^
21	192 ± 1.78	190.6 ± 1.81	130.4 ± 3.36^∗∗^	196.6 ± 1.67^∗^	201.2 ± 1.92^∗∗^	204 ± 1.58^∗∗^

∗ represents *p* < 0.05, ∗∗ represents *p* < 0.01, and ∗∗∗ represents *p* < 0.001 when compared to the normal control group.

## Data Availability

Data from the manuscript can be available on reasonable request.

## References

[1] Sheen Y.-J., Hsu C.-C., Jiang Y.-D., Huang C.-N., Liu J.-S., Sheu W. H.-H. (2019). Trends in prevalence and incidence of diabetes mellitus from 2005 to 2014 in Taiwan. *Journal of the Formosan Medical Association*.

[2] GBD 2021 Diabetes Collaborators (2023). Global, regional, and national burden of diabetes from 1990 to 2021, with projections of prevalence to 2050: a systematic analysis for the global burden of disease study 2021. *The Lancet*.

[3] Cole J. B., Florez J. C. (2020). Genetics of diabetes mellitus and diabetes complications. *Nature Reviews Nephrology*.

[4] American Diabetes Association (2014). Diagnosis and classification of diabetes mellitus. *Diabetes Care*.

[5] Wu H., Yang S., Huang Z., He J., Wang X. (2018). Type 2 diabetes mellitus prediction model based on data mining. *Informatics in Medicine Unlocked*.

[6] Ling S., Brown K., Miksza J. K. (2020). Association of type 2 diabetes with cancer: a meta-analysis with bias analysis for unmeasured confounding in 151 cohorts comprising 32 million people. *Diabetes Care*.

[7] Zaghloul N., Awaisu A., Mahfouz A., Alyafei S., Elewa H. (2022). A 5-year trend in the use of sodium-glucose co-transporter 2 inhibitors and other oral antidiabetic drugs in a Middle Eastern country. *International Journal of Clinical Pharmacy*.

[8] Wakeman M., Archer D. T. (2020). Metformin and micronutrient status in type 2 diabetes: does polypharmacy involving acid-suppressing medications affect vitamin B12 levels?. *Diabetes, Metabolic Syndrome and Obesity*.

[9] Torres C. A., Zampini I. C., Nuñez M. B., Isla M. I., Castro M. P., Gonzalez A. M. (2013). In vitro antimicrobial activity of 20 selected climber species from the Bignoniaceae family. *Natural Product Research*.

[10] Wang D., Cai M., Wang T. (2020). Ameliorative effects of L-theanine on dextran sulfate sodium induced colitis in C57BL/6J mice are associated with the inhibition of inflammatory responses and attenuation of intestinal barrier disruption. *Food Research International*.

[11] Smeriglio A., Ragusa S., Monforte M. T., D’angelo V., Circosta C. (2019). Phytochemical analysis and evaluation of antioxidant and anti-acetylcholinesterase activities of *Euphorbia dendroides* L. (Euphorbiaceae) latex. *Plant Biosystems - An International Journal Dealing with all Aspects of Plant Biology*.

[12] Khang N. V. D., Dao D. T. H., Mai N. T. T., Le Quan T., Nhi N. T. Y. (2023). Cytotoxicity, anti-diabeticity, and phytocomposition investigation of Vietnamese *Euphorbia tithymaloides* Linn.(Euphorbiaceae). *RSC Advances*.

[13] Wang Y., Yu X., Wang L., Zhang F., Zhang Y. (2020). Research progress on chemical constituents and anticancer pharmacological activities of *Euphorbia lunulata* Bunge. *BioMed Research International*.

[14] Ghauri M. A., Iqbal L., Raza A., Hayat U., Atif N., Javeed A. (2021). In vivo anti-inflammatory, antipyretic, analgesic activity and in vitro anti-proliferative activity of aqueous methanolic extract of *Euphorbia granulata* Forssk. *Future Journal of Pharmaceutical Sciences*.

[15] Patel D., Kumar R., Laloo D., Hemalatha S. (2012). Diabetes mellitus: an overview on its pharmacological aspects and reported medicinal plants having antidiabetic activity. *Asian Pacific Journal of Tropical Biomedicine*.

[16] Krishnaveni M., Mirunalini S., Karthishwa K., Dhamodhara G. (2009). Antidiabetic and antihyperlipidemic properties of *Phyllanthus emblica* Linn. (Euphorbiaceae) on streptozotocin induced diabetic rats. *Pakistan Journal of Nutrition*.

[17] Sharma N., Samarakoon K., Gyawali R. (2014). Evaluation of the antioxidant, anti-inflammatory, and anticancer activities of *Euphorbia hirta* ethanolic extract. *Molecules*.

[18] Zhu Q., Jiang M.-L., Shao F., Ma G.-Q., Shi Q., Liu R.-H. (2020). Chemical composition and antimicrobial activity of the essential oil From *Euphorbia helioscopia* L. *Natural Product Communications*.

[19] Mustafa I., Faisal M. N., Hussain G. (2021). Efficacy of *Euphorbia helioscopia* in context to a possible connection between antioxidant and antidiabetic activities: a comparative study of different extracts. *BMC Complementary Medicine and Therapies*.

[20] Mousavi L., Salleh R. M., Murugaiyah V., Asmawi M. Z. (2016). Hypoglycemic and anti-hyperglycemic study of *Ocimum tenuiflorum* L. leaves extract in normal and streptozotocin-induced diabetic rats. *Asian Pacific Journal of Tropical Biomedicine*.

[21] Khan D., Sharif A., Zafar M., Akhtar B., Akhtar M. F., Awan S. (2020). Delonix regia a folklore remedy for diabetes; attenuates oxidative stress and modulates type II diabetes mellitus. *Current Pharmaceutical Biotechnology*.

[22] Zafar M., Sharif A., Khan D. (2021). Preventive effect of *Euphorbia royleana* Boiss on diabetes induced by streptozotocin via modulating oxidative stress and deoxyribonucleic acid damage. *Toxin Reviews*.

[23] Abbas A., Hassan S. S. U., Sharif A., Ahmed S. (2020). Evaluation of the antioxidant and anti-inflammatory activities of solvent extracts of *Tricholepis chaetolepis* (Boiss) Rech. f. whole plant. *Natural Product Research*.

[24] Malik M., Sharif A., Hassan S. U. (2022). Amelioration of hyperglycaemia and modulation of pro-inflammatory cytokines by *Tamarix gallica* fractions in alloxan induced diabetic rats. *Archives of Physiology and Biochemistry*.

[25] Mousavi L., Salleh R. M., Murugaiyah V. (2018). Phytochemical and bioactive compounds identification of Ocimum tenuiflorum leaves of methanol extract and its fraction with an anti-diabetic potential. *International Journal of Food Properties*.

[26] Saleem U., Ahmad B., Ahmad M., Erum A., Hussain K., Irfan Bukhari N. (2016). Is folklore use of *Euphorbia helioscopia* devoid of toxic effects?. *Drug and Chemical Toxicology*.

[27] Ajiboye B. O., Ojo O. A., Akuboh O. S., Abiola O. M., Idowu O., Amuzat A. O. (2018). Anti-hyperglycemic and anti-inflammatory activities of polyphenolic-rich extract of *Syzygium cumini* Linn leaves in alloxan-induced diabetic rats. *Journal of Evidence-Based Integrative Medicine*.

[28] Qazi A. I., Ahmad B., Sahibzada M. U. K. (2023). Evaluation of antidiabetic activity of oxadiazole derivative in rats. *Evidence-Based Complementary and Alternative Medicine*.

[29] Toma A., Makonnen E., Mekonnen Y., Debella A., Adisakwattana S. (2015). Antidiabetic activities of aqueous ethanol and n-butanol fraction of *Moringa stenopetala* leaves in streptozotocin-induced diabetic rats. *BMC Complementary and Alternative Medicine*.

[30] Uyar A., Abdulrahman N. T. (2020). A histopathological, immunohistochemical and biochemical investigation of the antidiabetic effects of the Pistacia terebinthus in diabetic rats. *Biotechnic & Histochemistry*.

[31] Stalin A., Irudayaraj S. S., Gandhi G. R., Balakrishna K., Ignacimuthu S., Al-Dhabi N. A. (2016). Hypoglycemic activity of 6-bromoembelin and vilangin in high-fat diet fed-streptozotocin-induced type 2 diabetic rats and molecular docking studies. *Life Sciences*.

[32] Irudayaraj S. S., Stalin A., Sunil C., Duraipandiyan V., Al-Dhabi N. A., Ignacimuthu S. (2016). Antioxidant, antilipidemic and antidiabetic effects of ficusin with their effects on GLUT4 translocation and PPAR*γ* expression in type 2 diabetic rats. *Chemico-Biological Interactions*.

[33] Kim H. M. K. H. M., Kang J. S., Kim J. Y. (2010). Evaluation of antidiabetic activity of polysaccharide isolated from *Phellinus linteus* in non-obese diabetic mouse. *International Immunopharmacology*.

[34] Maoulainine L. B. M., Jelassi A., Hassen I., Ould O. M. S., Boukhari A. (2012). Antioxidant proprieties of methanolic and ethanolic extracts of *Euphorbia helioscopia*, (L.) aerial parts. *International Food Research Journal*.

[35] Ullah A., Munir S., Badshah S. L. (2020). Important flavonoids and their role as a therapeutic agent. *Molecules*.

[36] Rahimi-Madiseh M., Malekpour-Tehrani A., Bahmani M., Rafieian-Kopaei M. (2016). The research and development on the antioxidants in prevention of diabetic complications. *Asian Pacific Journal of Tropical Medicine*.

[37] Yang Y., Chen X., Luan F. (2021). *Euphorbia helioscopia* L.: a phytochemical and pharmacological overview. *Phytochemistry*.

[38] Yelumalai S., Giribabu N., Karim K., Omar S. Z., Salleh N. B. (2019). In vivo administration of quercetin ameliorates sperm oxidative stress, inflammation, preserves sperm morphology and functions in streptozotocin-nicotinamide induced adult male diabetic rats. *Archives of Medical Science*.

[39] Bae I.-S., Kim S. H. (2020). Sinapic acid promotes browning of 3T3-L1 adipocytes via p38 MAPK/CREB pathway. *BioMed Research International*.

[40] Yao Z., Li C., Gu Y. (2019). Dietary myricetin intake is inversely associated with the prevalence of type 2 diabetes mellitus in a Chinese population. *Nutrition Research*.

[41] Lalitha N., Sadashivaiah B., Talahalli R. R., Singh S. A. (2020). Lectin rich horsegram protein and myricetin activates insulin signaling–a study targeting PTP1*β*. *Journal of Functional Foods*.

[42] Vinayagam R., Jayachandran M., Xu B. (2016). Antidiabetic effects of simple phenolic acids: a comprehensive review. *Phytotherapy Research*.

[43] Amuri B., Maseho M., Simbi L., Okusa P., Duez P., Byanga K. (2017). Hypoglycemic and antihyperglycemic activities of nine medicinal herbs used as antidiabetic in the region of Lubumbashi (DR Congo). *Phytotherapy Research*.

[44] Razafindrakoto Z. R., Donno D., Tombozara N. (2020). Antioxidant, anti-inflammatory, and antidiabetic activities of leaves and stems of *Uapaca bojeri* Bail. (*Euphorbiaceae*), an endemic plant of Madagascar. *Pharmaceuticals*.

[45] Mustafa I., Anwar H., Irfan S., Muzaffar H., Ijaz M. U. (2022). Attenuation of carbohydrate metabolism and lipid profile by methanolic extract of *Euphorbia helioscopia* and improvement of beta cell function in a type 2 diabetic rat model. *BMC Complementary Medicine and Therapies*.

[46] Widharna R. M., Soemardji A. A., Wirasutisn K. R., Kardono L. B. S. (2010). Anti diabetes mellitus activity *in vivo* of ethanolic extract and ethyl acetate fraction of *Euphorbia hirta* L. herb. *International Journal of Pharmacology*.

[47] Guo X. X., Wang Y., Wang K., Ji B. P., Zhou F. (2018). Stability of a type 2 diabetes rat model induced by high-fat diet feeding with low-dose streptozotocin injection. *Journal of Zhejiang University Science B*.

[48] Saleem U., Ahmad B., Ahmad M., Hussain K., Bukhari N. I. (2014). Investigation of *in vivo* antioxidant activity of *Euphorbia helioscopia* latex and leaves methanol extract: a target against oxidative stress induced toxicity. *Asian Pacific Journal of Tropical Medicine*.

[49] Farhangi M. A., Javid A. Z., Dehghan P. (2016). The effect of enriched chicory inulin on liver enzymes, calcium homeostasis and hematological parameters in patients with type 2 diabetes mellitus: a randomized placebo-controlled trial. *Primary Care Diabetes*.

[50] Imo C., Arowora K. A., Ezeonu C. S. (2019). Effects of ethanolic extracts of leaf, seed and fruit of *Datura metel* L. on kidney function of male albino rats. *Journal of Traditional and Complementary Medicine*.

[51] Liu Z., Zeng Y., Hou P. (2018). Metabolomic evaluation of *Euphorbia pekinensis* induced nephrotoxicity in rats. *Pharmaceutical Biology*.

[52] Zhen R., Ban J., Jia Z., Liu Y., Li Z., Chen S. (2023). The relationship between non-HDL-C/HDL-C ratio (NHHR) and vitamin D in type 2 diabetes mellitus. *Diabetes, Metabolic Syndrome and Obesity*.

[53] Almalki D. A., Alghamdi S. A., al-Attar A. M. (2019). Comparative study on the influence of some medicinal plants on diabetes induced by streptozotocin in male rats. *BioMed Research International*.

[54] Saad E. A., Hassanien M. M., El-Hagrasy M. A., Radwan K. H. (2015). Antidiabetic, hypolipidemic and antioxidant activities and protective effects of *Punica granatum* peels powder against pancreatic and hepatic tissues injuries in streptozotocin induced IDDM in rats. *International Journal of Pharmacy and Pharmaceutical Sciences*.

[55] Elkotby D., Hassan A. K., Emad R., Bahgat I. (2018). Histological changes in islets of Langerhans of pancreas in alloxan-induced diabetic rats following Egyptian honey bee venom treatments. *International Journal of Pure and Applied Zoology*.

[56] Balamash K. S., Alkreathy H. M., Al Gahdali E. H., Khoja S. O., Ahmad A. (2018). Comparative biochemical and histopathological studies on the efficacy of metformin and virgin olive oil against streptozotocin-induced diabetes in Sprague-Dawley rats. *Journal of Diabetes Research*.

[57] Abdelrazek H., Kilany O. E., Muhammad M. A. A., Tag H. M., Abdelazim A. M. (2018). Black seed thymoquinone improved insulin secretion, hepatic glycogen storage, and oxidative stress in streptozotocin-induced diabetic male Wistar rats. *Oxidative Medicine and Cellular Longevity*.

[58] Wang J., Wang C., Li S. (2017). Anti-diabetic effects of Inonotus obliquus polysaccharides in streptozotocin-induced type 2 diabetic mice and potential mechanism via PI3K-Akt signal pathway. *Biomedicine & Pharmacotherapy*.

[59] Khin P. P., Lee J. H., Jun H. S. (2023). Pancreatic beta-cell dysfunction in type 2 diabetes. *European Journal of Inflammation*.

[60] Uxa S., Castillo-Binder P., Kohler R., Stangner K., Müller G. A., Engeland K. (2021). Ki-67 gene expression. *Cell Death & Differentiation*.

[61] Pei Y.-f., Yin X.-m., Liu X.-q. (2018). TOP2A induces malignant character of pancreatic cancer through activating *β*-catenin signaling pathway. *Biochimica et Biophysica Acta (BBA) - Molecular Basis of Disease*.

[62] Bologna-Molina R., Mosqueda-Taylor A., Molina-Frechero N., Mori-Estevez A. D., Sánchez-Acuña G. (2013). Comparison of the value of PCNA and Ki-67 as markers of cell proliferation in ameloblastic tumors. *Medicina Oral, Patologia Oral y Cirugia Bucal*.

[63] Berens H. M., Tyler K. L. (2011). The proapoptotic Bcl-2 protein Bax plays an important role in the pathogenesis of reovirus encephalitis. *Journal of Virology*.

[64] Strycharz J., Drzewoski J., Szemraj J., Sliwinska A. (2017). Is p53 involved in tissue-specific insulin resistance formation?. *Oxidative Medicine and Cellular Longevity*.

[65] Wang T., Wang J., Hu X., Huang X., Chen G. X. (2020). Current understanding of glucose transporter 4 expression and functional mechanisms. *World Journal of Biological Chemistry*.

[66] Chen H., Xiong L., Wang N. (2018). *Chimonanthus nitens* Oliv. leaf extract exerting anti-hyperglycemic activity by modulating GLUT4 and GLUT1 in the skeletal muscle of a diabetic mouse model. *Food & Function*.

[67] Hemmati M., Serki E., Gholami M., Hoshyar R. (2017). Effects of an ethanolic extract of Berberis vulgaris fruits on hyperglycemia and related gene expression in streptozotocin-induced diabetic rats. *Clinical Phytoscience*.

[68] Asghar A., Sharif A., Awan S. J. (2023). *Ficus johannis* Boiss. leaves ethanolic extract ameliorate streptozotocin-induced diabetes in rats by upregulating the expressions of GCK, GLUT4, and IGF and downregulating G6P. *Environmental Science and Pollution Research*.

[69] Pari L., Chandramohan R. (2017). Modulatory effects of naringin on hepatic key enzymes of carbohydrate metabolism in high-fat diet/low-dose streptozotocin-induced diabetes in rats. *General Physiology and Biophysics*.

[70] Kang H. S., Cho H. C., Lee J. H. (2016). Metformin stimulates IGFBP-2 gene expression through PPARalpha in diabetic states. *Scientific Reports*.

